# Evaluation of the reported data linkage process and associated quality issues for linked routinely collected healthcare data in Multimorbidity research: a systematic review.

**DOI:** 10.23889/ijpds.v7i3.2047

**Published:** 2022-08-25

**Authors:** Maria Elstad, Abdel Douiri, Jo Røislien

**Affiliations:** 1 King's College London; 2 University of Stavanger

## Objectives

The objective for this systematic review was to examine how the record linkage process was reported and to understand challenges related to accessing, linking, and analysing linked routinely collected data used for multimorbidity research.

## Approach

Twenty studies were included, of which seventeen looked at the relationship between two specified long-term conditions. Fourteen studies received the linked dataset from an external data linkage provider. Hospital Episode Statistics was the most common source of data (n=5). Eight studies reported variables used for the data linkage, while only two studies reported pre-linkage checks. The quality of the linkage was assessed by three studies, of which two reported linkage rate and one reported raw linkage figures. Only one study checked for bias by comparing patient characteristics of linked and non-linked records.

The findings from this study will feed into further guidance to understand and minimise bias due to linkage error in medical research.

## Results

Twenty studies were included, of which seventeen looked at the relationship between two specified long-term conditions. Fourteen studies received the linked dataset from an external data linkage provider. Hospital Episode Statistics was the most common source of data (n=5). Eight studies reported variables used for the data linkage, while only two studies reported pre-linkage checks. The quality of the linkage was assessed by three studies, of which two reported linkage rate and one reported raw linkage figures. Only one study checked for bias by comparing patient characteristics of linked and non-linked records.

## Conclusion

UK LLC provides a strategic research-ready platform for longitudinal research: a clear step change from pre-pandemic capability. With sustained investment, and through exploring options to extend linkages and generalise to wider purposes, UK LLC is positioned to inform cross-cutting themes such as understanding health and social inequalities, health-social-environmental interactions, and managing the COVID-19 recovery.

